# TET1 Isoforms Have Distinct Expression Pattern, Localization and Regulation in Breast Cancer

**DOI:** 10.3389/fonc.2022.848544

**Published:** 2022-05-12

**Authors:** Mahmoud Alzahayqa, Abrar Jamous, Areej A. H. Khatib, Zaidoun Salah

**Affiliations:** ^1^Molecular Genetics Lab, Medicare Laboratories, Ramallah, Palestine; ^2^Department of Molecular Biology and Biochemistry, Al Quds University, Jerusalem, Palestine; ^3^Women Health Research Unit, McGill University Health Center, Montreal, QC, Canada; ^4^Molecular Genetics and Genetic Toxicology Program, Arab American University, Ramallah, Palestine

**Keywords:** TET1, methylation, isoforms, breast cancer, demethylation

## Abstract

TET1 regulates gene expression by demethylating their regulatory sequences through the conversion of 5-methylcytosine to 5-hyroxymethylcytosine. TET1 plays important roles in tissue homeostasis. In breast cancer, TET1 was shown to play controversial roles. Moreover, TET1 has at least two isoforms (long and short) that have distinct expression pattern and apparently different functions in tissue development and disease including breast cancer. We hypothesized that TET1 isoforms have different expression patterns, localization and regulation in different types of breast cancer. To prove our hypothesis, we studied the expression of TET1 isoforms in basal and luminal breast cancer cell lines, as well as in basal and luminal breast cancer animal models. We also studied the effect of different hormones on the expression of the two isoforms. Moreover, we assessed the distribution of the isoforms between the cytoplasm and nucleus. Finally, we overexpressed the full length in a breast cancer cell line and tested its effect on cancer cell behavior. In this study, we demonstrate that while Estrogen and GnRH downregulate the expression of long TET1, they lead to upregulation of short TET1 expression. In addition, we uncovered that luminal cells show higher expression level of the long isoform. We also show that while all TET1 isoforms are almost depleted in a basal breast cancer animal model, the expression of the short isoform is induced in luminal breast cancer model. The short form is expressed mainly in the cytoplasm while the long isoform is expressed mainly in the nucleus. Finally, we show that long TET1 overexpression suppresses cell oncogenic phenotypes. In conclusion, our data suggest that TET1 isoforms have distinct expression pattern, localization and regulation in breast cancer and that long TET1 suppresses oncogenic phenotypes, and that further studies are necessary to elucidate the functional roles of different TET1 isoforms in breast cancer.

## Introduction

Cancer is a genetic disease that is characterized by abnormal gene expression and function. It arises as a result of either genetic mutations in oncogenes and tumor suppressor genes or epigenetic alterations that modulate gene expression. Collectively, these events abrogate cell homeostasis and lead to tumor formation and progression. One of the most important epigenetic changes that play a role in carcinogenesis is DNA methylation. Aberrant DNA methylation in cancer cells alters the expression of genes that are responsible for cancer hallmarks (1, 2). Moreover, specific cancer cell methylation patterns that distinguish them from normal cells were identified (3, 4). While DNA methylation mechanisms are well understood and documented, mechanisms responsible for counterbalancing and reversing methylation were elusive until the discovery of Ten Eleven Translocation proteins (TETs) (5).

TET family includes three members; TET1, TET2 and TET3. These enzymes are hydroxylases that lead to DNA demethylation through successive oxidation reactions (6, 7). TET enzymes have common as well as distinct functions in embryonic development, stem cell biology, cell differentiation as well as neuronal cell biology (8). In cancer context, TET enzymes were shown to be involved in liquid as well as solid tumors (9–13). In specific, TET1 was also shown to play different roles in different types of cancers including, liver, cervical, gastric, colon and breast cancers (14–18).

In breast cancer, TET1 has been identified as both a tumor suppressor as well as an oncogene. For example, TET1 promoter is methylated in different types of cancers, including breast cancer (19). In the same paper, the authors demonstrated that TET1 catalytic domain overexpression reverses the methylation pattern of different tumor suppressor genes and leads to inhibition of cancer cell tumorigenic potential (19). In addition, it was found that reduced expression of TET1 is associated with poor prognosis of early breast cancer patients (20). This finding was supported also by a recet meta analysis and literature review (21). Moreover, another study showed cytoplasmic mislocalization of TET1 and thus decreased 5hmC levels correlates with poor prognosis especially in PR and ER negative breast cancer patients (22) Mechanistically, TET1 was shown to be antitumorigenic by regulating different targets. In mouse xenografts, TET1 suppresses tumor growth and metastasis by regulating the HMGA2-TET1-HOXA9 pathway, which is implicated in the epigenetic regulation of human breast cancer. In this article, it was shown that HMG2 depletion leads to TET1 expression induction that induces HOXA and its downstream genes (23). In a different study, TET1 was shown to inhibit tumor invasion and metastasis in part by maintaining higer expression of TIMP2 or TIMP3 (24). In triple negative breast cancer (TNBC), EZH2 was shown to downregulate TET1 expression and consequently inhibited tumor senesence and apoptosis, indicating that TET1 is a tumor suppressor, also in TNBC (25).

TET1 oncogenic function was also demonstrated in different publications. TET1 was shown to be overexpressed in breast cancer and that its knockdown leads to inactivation of PI3K-mTOR pathway (26). Also, in a recent study, TET1 was shown to maintain cancer stem cells in TNBC (27). Moreover, FECR1 [a circular RNA of Friend leukemia virus integration 1 (FLI1)], was shown to recruit TET1 to the promoter of FLI1 oncogene, which leads to promoter hypomethylation and FLI1 oncogene overexpression in breast cancer cells (28). In addition, TET1 was shown to be overexpressed in basal like breast cancer samples and that its level negatively correlates with immune defense mechanisms that are related to NF-kB signaling pathway (29).

TET1 has at least two isoforms; a long isoform that retains the CXXC domain that binds to DNA and a shorter isoform that lacks this domain. These two isoforms are generated by the usage of two alternative transcription start sites. The short isoform was found to be differentially expressed in somatic tissues (30). Also, the expression of the short isoform was shown to be cell context dependent. For example, it was shown to be expressed in poorly differentiated gonadotropes (31). In breast cancer, mainly in TNBC, the short isoform was found to be overexpressed and correlates with worse overall survival (32). It was shown also, by the same group, that the short isoform activates the PI3K pathway in a subset of TNBC (26).

While plenty of evidence has shown that TET1 is a tumor suppressor gene, not less evidence has proved that TET1 supports oncogenic changes in breast cancer, especially after the discovery of the short isoform of TET1. This controversy in the published literature made it necessary to try to better understand the expression pattern and regulation of the different TET1 isoforms in breast cancer. In the current work we demonstrate the presence of a heterogenous expression level and localization of TET1 in breast cancer samples. We also show that the two TET1 isoforms are regulated differently with hormones, and that their localization is different, and that their expression level varies between luminal and basal cell lines and animal models.

## Materials and Method

### Cell Culture

Breast cancer cell lines including MCF7, MDA MB231, T47D, HCC70, Sum 149, BT 549 cells, and Human Embryonic Kidney (HEK 293T) cells were grown in RPMI media (Gibco-Thermofisher), supplemented with 10% FBS (Gibco-Thermofisher), 1% glutamine, and 1% penicillin/streptomycin (Biological Industries). MCF10A cells were grown in DMEM/F12 media (Biological industries) supplemented with 5% horse serum (Biological industries), 1% glutamine, 1% Penicillin/Streptomycin (Biological industries), 20 ng/mL EGF, 10 μg/mL insulin, 0.5 μg/mL hydrocortisone, and 100 ng/mL cholera toxin (Sigma Aldrich). All Cells were incubated in humidity chamber on 37°C with 5% CO2.

### RNA Extraction, cDNA Synthesis and qRT-PCR

Tri reagent (Sigma Aldrich) was used for RNA extraction following manufacturer’s protocol. DNA contamination was avoided by using DNase I treatment kit (biolabs). cDNA was synthesized using Q-Script cDNA synthesis kit according to manufacturer instructions (Quanta Biosciences). Relative quantitative real-time PCR (qRT-PCR) was performed using Applied Biosystems^®^ 7500 Real-Time PCR machine using power SYBR Green Mix (Applied Biosystems). In order to check for the presence of different TET1 isoforms, we used exon specific primers designed to target specific human TET1 exons and mouse Tet1 exons according to published primer sequences (31, 32) respectively. For exon specific expression analysis, the relative TET1 isoform expression was in relation to TET1 exon 10. Other primers used are listed in **Supplementary Table 1**.

### Clinical Samples and Immunohistochemistry (IHC)

A total of 50 breast cancer and corresponding adjacent normal tissue samples were collected From Medicare laboratories, Ramallah Palestine. Ethical approval was obtained from Helsinki committee. (approval no. PHRC/HC/588/19). Our study was conducted in accordance with approved guidelines. 5 µm tumor sections were deparaffinized and rehydrated with Xylene (LOBA CHEMIE) 3 times for 5 min, then 100% ethanol (BioLab) 3 times for 2 min, 95% ethanol 2 times for 2 min, 80% ethanol for 3 min, and then transferred into distilled water (DW) for 5 min. After that, tissues were denatured for 4 min in pressure cooker in citrate buffer (Sodium citrate dehydrate and Citric acid, both Sigma-Aldrich) 0.01 mol/L, pH 6.0. Then slides were cooled to RT in a cold water bath, and then washed and incubated with DW for 5 min. Afterwards slides were blocked by 3% Hydrogen peroxidase (Sigma-Aldrich), washed and incubated with DW for 5 min. After that, slides were blocked again with CAS^®^ block (Invetrogen,00-8120) reagent for 15 min. Then the sections were incubated with anti-TET1 Abs (TET1 191698, Abcam) overnight at 4°C according to manufacturer’s recommendations. Next, slides were washed 3 times for 5 min each with X1 TTBS (100 ml TBS X10, 900 mL DW, and 0.5 mL Tween, Sigma-Aldrich). Then, slides were incubated with secondery antibody conjugated to HRP (Bethyl, A120-101P). To visualize the stained tissue, slides were incubated with HRP chromogen substrate (Impress HRP reagent kit, vector laboratories). Finally, slides were counterstained with Mayer’s hematoxylin (BioGnost).

### Subcellular Fractionation and Western Blot Analysis

Nuclear and cytoplasmic protein fractions were prepared as described previously (33) In brief, cells were scraped in PBS, and after centrifugation, the cell pellet was reconstituted in a hypotonic lysis buffer [10 mmol/L HEPES (pH 7.9), 10 mmol/L KCl, 0.1 mmol/L EDTA] supplemented with 1 mmol/L DTT and a broad-spectrum cocktail of protease inhibitors (Sigma-Aldrich). The cells were allowed to swell on ice for 15 min, then NP40 was added, and cells were lysed by vortex. After centrifugation the cytoplasmic fraction in the supernatant was collected, and nuclear extracts were obtained by incubating nuclei in a hypertonic nuclear extraction buffer [20 mmol/L HEPES (pH 7.9), 0.42 mol/L KCl, 1 mmol/L EDTA] supplemented with 1 mmol/L DTT for 15 min at 4°C. Protein concentration was quantified using Bradford protein assay (BioRad.) Samples were run on polyacrylamide gel (Biological industries), transferred to Nitrocellulose membrane by SD semi-dry transfer cell (BioRad). Membranes were blocked with skim milk (Sigma) and immunoblotted with primary antibody (anti-TET1 GT 1462, Sigma). Afterwards membranes were washed and blotted with secondary anti-mouse horse raddish peroxidase conjugated antibody (Bethyl, A90-109P) according to the manufacturer’s instructions. Signal was visualized using Western Protein ECL substrate (Thermofisher), and G: BOX Chemi XX6 Gel Imaging System (Geneflow).

### Lentivirus Preparation

Lentivirus particles were generated by three plasmid expression system, in which HEK 293T cells were co-transfected with the following three vectors: packaging GAG- pol (Addgene), envelope pCMV-VSV-G (Addgene), and TET1-PSF Lenti or PSF lenti vector. One day before the transfection, HEK-293T cells were plated to be 60% confluent. On the next day, cells were fed with fresh medium and transfected using MIRUS (TransfectionExperts, MIR2300) transfection reagent according to manufacturer instructions. Briefly, 2.2 µg packaging GAG-Pol, 1.2 µg Envelop VSVG, and 5 µg of each TET1-PSF-lenti and PSF-lenti vector were transferred to tubes containing 21 µl MIRUS mixed with 2ml serum free medium. After 15 min incubation at RT the mixture was dropped on cell culture media. 24 hrs after transfection, cell culture media was changed with a fresh media. 2 and 3 days after transfection, cell culture media containing the viral particles was collected and centrifuged for 10 min at 53000 rpm to get rid cellular debris. Finally, the collected virus containing media were centrifuged at 40000 rpm for 2 hrs using BECKMAN COULTER (OptimaTM LE-80K Ultracentrifuge). After centrifugation, most of the media was removed and the viral particles were then resuspended and filtered using 0.45 μm filters (JET BIOFIL).

### Infection and Selection

0.5 million MDA MB231 cells were infected with 0.75 mL of viral particles suspension. Cells were incubated with viral particles for two days in CO2 incubator at 37°C. Next, media containing viruses was removed, and replaced with refresh one for one day. We repeated the infection procedure twice. To select for clones, cells were grown in media containing 1 µg/mL of puromycin (Sigma) untile control un infected cells completely died.

### Cell Count

3x10^4^ cells were seeded in 6 well plate in triplicates and cells were counted over 3 days as follows. Cells were first trpsynized and collected into 15 ml conical tubes and centrifuged at 1600 RPM for 10 min. Then the supernatant was removed and cells were re-suspended in 1 mL media. Next, 10 µl of the homogenous supernatant was counted using counting chamber slides.

### XTT Test

2 X10^3^ cells were seeded in triplicate in 96 well plate and cell proliferation was assessed over 3 days using XTT kit (Biological industries) according to the manufacturer’s instructions. Results were read using ELISA reader, (BioTek EL-X800).

### Wound Healing

2.5 x10^5^ cells were seeded in triplicates in 12 well plates. Upon reaching 100% confluency, cell monolayer was scratched using the 10 µl plastic pipette tip. After removing floating cells, the same area in plate was photographed over the needed period of time using a camera attached to Inverted Microscope (Olympus, CK-40).

### Survival Assay

200 cells were seeded in triplicates in 6 well plate. Each 3-4 days, the media was changed until cell colonies were visible by naked eye. Then the media was removed and wells were washed with PBSX1 (Biological industries). Then PBS was aspirated and wells were left to dry. After drying, cells were fixed with absolute methanol (Sigma) for about 15 min at RT. Afterwards, wells were left to dry and then stained using Coomassie blue (Bio-Rad) for about 15 min. Finally, the stain was removed and wells were washed using tap water.

## Results

### TET1 Enzyme Expression in Breast Cancer Tissue Samples

**Figure 1:**
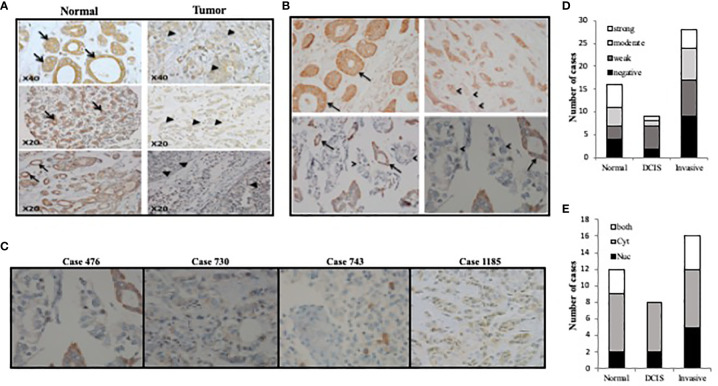
TET1 expression pattern in breast cancer samples. **(A)** Representative images of TET1 IHC staining showing loss or weak staining of TET1 in malignant cells (right panel, arrow heads) compared to benign or normally appearing cells of mammary tissue alveoli or ducts (left panel, arrows). **(B)** Correlation between TET1 expression and differentiation level in breast cancer samples. Representative images of TET1 IHC staining showing loss or decreased levels of TET1 expression in poorly differentiated (arrow heads) compared to well differentiated tissue (arrows). **(C)** Representative images of TET1 IHC staining showing different expression level ranging from heterogeneous (case 476), weak (case 730), negative (case 743), and mixed cytoplasmic and nuclear (case 1185) staining. Brown color indicates positive staining for TET1. **(D, E)** Statistical analysis of IHC staining showing the heterogeneity of TET1 staining strength and distribution in different tissue samples.

To test the expression pattern of TET1 in breast cancer in patient samples, we stained different types of human breast cancer samples by IHC using antisera against TET1 enzyme. Distinct TET1 expression levels and localization were seen in normal and tumor tissue, or when comparing tumors from different patients. For example, as shown in **Figure 1A**, in normally appearing tissue, more than 50% of the samples show moderate to strong expression while the percentage of moderate to strong expression is about 15% and 35% in DCIS and invasive tumors respectively (**Figures 1A, D**). Moreover, we noticed that the localization of TET1 in the nucleus and cytoplasm is also heterogeneous. Some samples showed exclusive either nuclear or cytoplasmic expression, while others showed both nuclear and cytoplasmic expression (**Figure 1**). Of note, the percentage of samples that displayed nuclear expression was higher in DCIS and highest in invasive tumors (**Figure 1E**). In addition, we noticed that the expression level of TET1 correlates with the degree of differentiation of the tumor. Positive brown staining is evident in well-differentiated tumors (**Figure 1B** arrows), which is lost when the same tumor progresses towards a poorly differentiated one (**Figure 1B** arrow heads). Moreover, the expression pattern of TET1 enzyme varied between tumors obtained from different patients (**Figure 1C**). These results indicate that TET1 expression is very heterogeneous in breast cancer samples and its expression correlates at least with breast cancer cell differentiation.

### Effect of GnRH and Estrogen on the Expression of TET1 Isoforms

**Figure 2:**
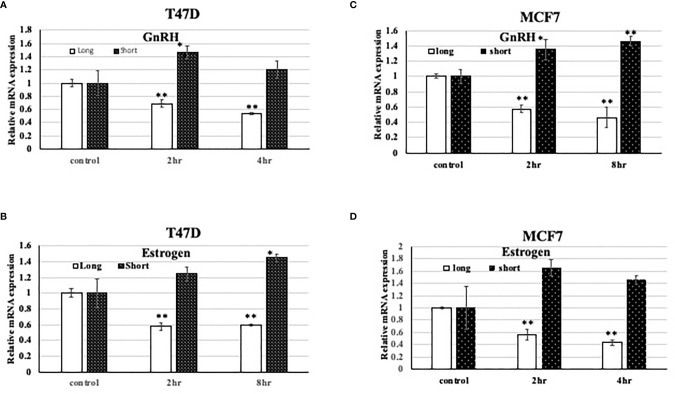
Effect of hormone treatment on the expression pattern of TET1 isoforms in the breast cancer cell line T47D. **(A–D)**. qPCR results on different TET1 isoforms after treating cells with the indicated hormone for the indicated period of time. The mRNA levels are shown after normalization to the level of the housekeeping gene UBC and relative to mRNA levels in control untreated cells. Bars represent SD of the mean of three replicates. * and ** indicated p value < 0.05 and 0.01 respectively.

In different research articles (31, 34), it was demonstrated that TET1 expression changes or is associated with hormone levels. However, the effect of different hormone treatment on different TET1 isoforms in breast cancer was not tested. Here, we tested the effect of both GnRH and Estrogen (E2), on the expression of both, the long and short isoforms of TET1 in both T47D and MCF7 cells. As shown in **Figure 2**, both hormone treatments have different effects on the expression pattern of the long and short isoforms. Both hormone treatments lead to full length TET1 mRNA down regulation but they mainly lead to statistically significant increase in the expression of the short isoform. As shown in **Figure 2**, the pattern is similar with the two cell lines used in this experiment. These results show that hormone treatments have different effects on the expression of full length and short TET1 isoforms.

### Differential TET1 Isoform Expression Between Basal and Luminal Cell Lines and Isoform Specific Cellular Localization

**Figure 3:**
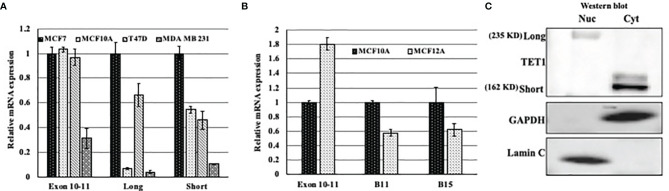
Expression pattern of TET1 isoforms in breast cancer cell lines. **(A)** qPCR results of different TET1 isoforms in different cell lines. **(B)** qPCR results of different TET1 isoforms in immortalized non-transformed mammary gland cells representing basal (MCF10A) and luminal (MCF12A) cell-of-origin. In both experiments, primers flanking Exons 10 and 11 were used as our reference point. The mRNA levels are shown after normalization to the level of the housekeeping gene UBC. Bars represent standard error of the mean of three replicates. **(C)** Western blot analysis showing the distribution of the long and short isoforms between nuclear (Nuc) and Cytoplasmic (Cyt) cell protein fractions.

Previous work has shown that TET1 has different isoforms; one long isoform and at least two other short isoforms. In this study we tried to find if there is a correlation between the expression of different isoforms and cell-of-origin of breast cancer cell lines (basal versus luminal). To this end, we tested the expression level of the isoforms in the luminal cell lines MCF7 and T47D, and basal cells MCF10A and MDA MB231. After treating RNA with DNase, we quantitated the expression of TET1 using primers that target either the long or the short isoforms of TET1. The expression level of Exons 10 and 11 was used as our reference point. As shown in **Figure 3A**, all cells express higher levels of the shorter TET1 isoform regardless the cells being basal or luminal. However, our results show that luminal cells express higher levels of the longer TET1 isoform compared to very low expression levels of this isoform in basal cells. Moreover, we measured TET1 expression in non transformed basal and luminal (MCF10A and MCF12A respectively) mammary epithelial cells. Our results show that while MCF12A cells show higher expression level of TET1 as measured by exon 10-11 expression (**Figure 3B**), MCF10A cells which are basal cells, show higher expression of TET1 short isoforms (**Figure 3B**). After showing the heterogeneity of TET1 isoform expression, we wanted to test their localization in the cell. To this end, we prepared cytoplasmic and nuclear protein fractions and tested TET1 expression in Western blot analysis. Our results show that the long isoform is expressed mainly in the nucleus, and that the short isoform is expressed mainly in the cytoplasm in MCF7 cells (**Figure 3C**). All together, these results show a heterogenous expression pattern of the different isoforms between basal and luminal breast cancer cell lines and that the two isoforms have different cellular localization in MCF7 cell line.

### The Expression Pattern of the Short *Tet1* Isoform in Different Breast Cancer Animal Models

**Figure 4:**
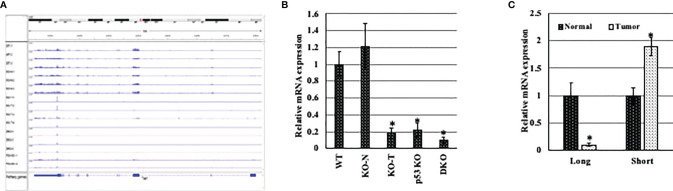
Expression pattern of different *Tet1* isoforms in different breast cancer animal models. **(A)** Visual presentation of RNA seq data for RNA extracted from conditional mammary gland *p53* and *Wwox* single and double knockout (w-KO: *Wwox* knockout, p-KO: *p53* knockout, D-KO: double knockout for both *p53* and *Wwox*) mice. RNA obtained from normal mammary gland tissue isolated from wild type mice (WT) or from pre-cancerous tissue of D-KO mice (N-KO) were used as control samples. **(B)** qRT-PCR on RNA extracted from conditional mammary gland *p53* and *Wwox* single and double knockout (w-KO: *Wwox* knockout, p-KO: *p53* knockout, D-KO: double knockout for both *p53* and *Wwox*) mice. RNA obtained from normal mammary gland tissue isolated from wild type mice (WT) or from pre-cancerous tissue of D-KO mice (N-KO) were used as control samples. **(C)** qRT-PCR on RNA extracted from mouse mammary tumors (black bars) and normal tissue (white bars), after DNase treatment, for Exons 1 (represents the longer *Tet1* isoform) and 1.5 of *Tet1* (represents the shorter isoform). The mRNA levels are shown after normalization to levels of *Rpl0*. Bars represent SEM, n=6. * indicates that p-value is <0.05.

To elucidate whether the presence of a specific *Tet1* isoform is relevant to breast tumorigenesis, we tested the expression of the short and full length *Tet1* in normal and tumor mammary tissues obtained from either *p53* and *WWOX* single and double knockout mice, which give rise to basal triple negative mammary gland tumors (from Prof. Rami Aqeilan, Hebrew University) or from an MMTV-PyMT transgene mouse model that give rise to luminal tumor type (RNA provided by Dr. Itay Ben-Borath, Hebrew university). In the first model (basal tumors), we noticed that in *Wwox* (w-KO), *p53* (p-KO) and *Wwox* and *p53*(D-KO) knockout mice there is a drastic reduction in the expression level of *Tet1* in all tumors tested compared to normal mammary gland tissue isolated from either wild type mice (wt) or knockout mice (N-KO), without preferential expression of any of *Tet1* exon (**Figures 4A, B**), indicating that all TET1 isoforms were downregulated, which might indicate that all *TET1* isoforms can be deleted in specific tumor types. In the MMTV-Py-MT model (luminal tumors), qRT-PCR revealed low levels of exon 1 (Tet1 long isoform) in tumor tissue and a twofold increase expression level of the shorter *Tet1* gene in tumor tissue suggesting the possible relevance of this isoform in breast tumorigenicity in specific tumor types (**Figure 4C**).

### Tumor Suppressor Function of Full Length TET1

**Figure 5:**
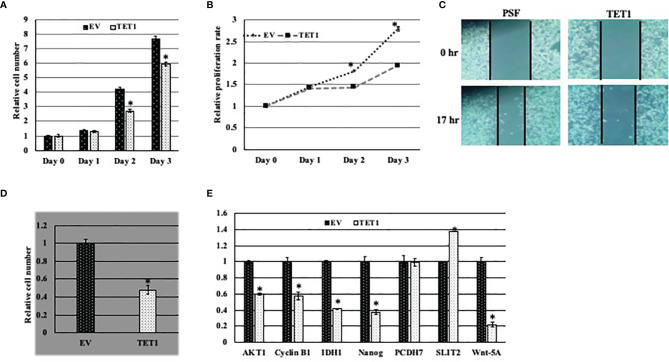
Effect of TET1 overexpression on MDA MB231 cell phenotypes. Representative graph showing the relative proliferation rate of TET1 overexpressing cells compared to control cells using cell count assay **(A)** and XTT assay **(B)**. Cell growth was monitored over three days and cell growth is blotted in reference to day 0. **(C)**. Representative images showing the migration capability of TET1 overexpressing cells in comparison to control cells using wound healing assay. Cell migration was monitored after 17 hrs. **(D)**. Representative statistical analysis of the survival rate of TET1 overexpressing cells in comparison to control cells using cell survival assay. **(E)** Relative qRT-PCR results showing the expression level of different TET1 target genes in MDA MB231 cell line. mRNA levels are shown after normalization to the level of the housekeeping gene *hUBC* and relative to mRNA level of each gene in MDA MB231 infected with EV. All experiments were done in triplicates. Bars represent SEM. * indicates that p-value is <0.05.

In order to study the function of at least the full TET1 enzyme in breast cancer, we infected breast cancer cell line MDA MB231 with viral particles either expressing HA-tagged full length *TET1* (TET1-Lenti) or empty viral vector control (PSF-Lenti). We first confirmed the overexpression of FL-TET1 in the cells by qRT-PCR (**Figures S1A, B**). The overexpression was further confirmed with Western blot analysis (**Figure S1C**). Then we tested the effect of long-TET1 overexpression on different cancer cell hallmarks including cell proliferation, migration and survival. To test TET1 overexpression effect on cell proliferation, we compared the growth rate of TET1 overexpressing cells to the growth rate of control cells using both cell count and xtt assays. In both assays, TET1 overexpression inhibited cell proliferation by about 35% compared to control cells (**Figures 5A, B**). Another cancer cell hallmark that we tested is cell migration. To evaluate TET1 overexpression on cell migration, we performed wound healing assay. As it appears in **Figure 5C**, TET1 overexpression inhibited cell migration capacity by about 20% in comparison to control cells. Cell autonomy and survival independence on cell-cell communication is a hallmark that characterizes cancer cell growth. To elucidate the effect of TET1 manipulation on cell survival, we did cell survival assay by culturing a few number of cells over a big surface area. In comparison to control cells, TET1 manipulated cells showed a lower cell survival index. The survival index was lowered by approximately 50% (**Figure 5D**). To explain the phenotypes related to TET1 overexpression at the molecular level, we tested the effect of TET1 manipulation on the expression level of different genes that are linked to the tested cancer hallmarks. We tested the expression of both oncogenes and tumor suppressor genes in MDA MB231 cells infected with either empty vector (PSF) or TET1(TET1) lentiviral vector. As expected, some genes didn’t show any significant change in their expression, while the expression of others was either induced or reduced upon TET1 overexpression. Interestingly, genes behaved as expected in correlation with TET1 suppressive function, ie; tumor suppressor genes either didn’t change (*PCDH7*) or were induced (*SLIT2*) (**Figure 5E**), while oncogenes were repressed (*IDH1*, *Cyclin B1*, *Nanog*, *AKT1*) (**Figure 5E**).

## Discussion

The role of TET1 in breast cancer is controversial. While some studies indicated that TET1 is a tumor suppressor gene (20, 21), others demonstrated that it is an oncogene (16, 32). In the current study our results indicate that the picture seems to be more complicated and that the role of TET1 in breast cancer is not black and white. We show that the expression pattern of TET1 in breast cancer tissue samples is very heterogeneous in the sense of expression level and localization. In this sense, our results are in agreement with different studies that have shown that TET1 expression is variable between different subtypes of breast cancer or in the same subtype of cancer that have specific molecular markers (26, 29). For example, the expression of TET1 was shown to negatively correlate with miR-29a and this negative correlation was stronger in estrogen receptor (ER) negative breast cancer samples compared to ER+ ones (35) In another research that studied the role of TET1 in TNBC, TET1 expression was shown to inversely correlate with the expression of EZH2 (25). Also, TET1 was shown to inhibit cancer invasion and metastasis and correlates with better survival (Cell Reports 2, 568–579, September 27, 2012). In these studies, TET1 was proved to be a TSG. On the other hand, TET1 was shown to have oncogenic functions. For example, TET1 expression pattern in tissue samples was shown to correlate with poor prognosis in breast cancer patients (36) and that it’s expression is elevated and correlates with the hypoxic level in breast cancer samples (36). Also, our results demonstrate a correlation between the differentiation level of the tumor and TET1 level, where high level of TET1 accompanies higher level of differentiation. In fact previous studies have proved that TET1 level correlates with the differentiation level of tissues in different contexts (30, 31).

Regarding TET1 distribution between the nucleus and cytoplasm, it is noted that not all previous studied pointed to the fact that TET1 is distributed between the cytoplasm and nucleus. Although this fact was neglected in many published papers that studied the role of TET1 in breast cancer, it appears that the distribution pattern of TET1 can affect its catalytic function. This was demonstrated in two independent studies that investigated the role of TET1 in gastric and breast cancers (18, 22). In these studies, the authors revealed that “TET1cytoplasmic mislocalization” correlates with 5hmC depletion and worse disease outcomes (18, 22). These findings raise the following questions. First, Is cytoplasmic TET1 not functional because it does not access the nucleus (sequestered in the cytoplasm)? Second, Which isoform is expressed in the cytoplasm and which one is in the nucleus? In our study we found that, at least in MCF7 cell line, that the short form is cytoplasmic while the long isoform is nuclear. Another missing piece in previous studies that looked at TET1 expression in tissue samples, using IHC, is that some of them were done before the discovery of the short TET1 isoform and others used anti-TET1 antibodies that detect TET1 C-terminus (18, 22), which can miss the detection of long length TET1 enzyme. These limitations can lead to immature conclusions about TET1 isoform roles in breast cancer, especially in light the fact that different TET1 isoforms might have different functions, expression patterns and cellular localization.

Because we noted that TET1 isoforms behave differentlt, and because breast cancer behavior is affected by hormones, we studied the effect of different hormones on the expression of different TET1 isoforms. Different articles have shown that TET1 expression is regulated by GnRH and steroids (31, 34). These studies proved that TET1 expression by hormones is cell- and context- specific, and that TET1 isoforms are differentially expressed upon hormone treatment (31). This distinct pattern of regulation of the two isoforms supports our current findings where we see that the long and short TET1 isoforms are reciprocally regulated by Estrogen and GnRH hormones.

Breast cancer is very heterogeneous, and it is believed, at least in part, that different cell-of-origin of tumors leads to tumor heterogeneity and discrete tumor behavior and clinical outcomes. In breast cancer, there are at least six main molecular tumor subtypes, where some of which are luminal tumors that have relatively better outcomes, and basal tumors that have unfavorable prognosis (37, 38). Here we demonstrate that in the basal cell lines the long isoform expression is very low compared to luminal cells. This pattern of expression might indicate that at least in the tested cell lines the loss of full length of TET1 is more important than the overexpression of the short isoform especially if take into consideration that the aggressive basal cell line MDA MB231 expresses the lowest level of both short and long isoforms. Of course, this conclusion is an indication that the expression level and pattern of TET1 isoforms is different between different types of breast cancer. However, this conclusion cannot be generalized because these are only cell lines that don’t recapitulate what takes place in the tumor where the gene expression level of different genes can be affected by tumor microenvironment. Thus, to further shed light on the correlation between tumor subtype and TET1 isoform expression pattern, we tested the expression level of TET1 isoforms in basal and luminal breast cancer animal models. In the basal tumor animal model that gives rise to triple negative breast cancer tumors, we see very low levels of both long and short isoforms. These results are in concordance with our results that we obtained with the triple negative breast cancer cell line MDA MB231 where we see low expression of both TET1 isoforms. However, these results are in dis concordance with previous studies that showed that the short isoform is overexpressed in TNBC (32). On the other hand, in the luminal model, we show that the expression of the long isoform is down regulated while that of the short isoform is upregulated. These results are in agreement with the notion that the long isoform has tumor suppressor functions (L. 20). This suppressor function is supported by our results in the current study where we demonstrate that long TET1 over expression suppresses oncogenic phenotypes. Of course, these results don’t preclude that the short isoform is important in the pathogenesis of TNBC but indicates that further studies are needed in order to elucidate the different functions of the TET1 isoforms in different breast cancer types.

In this study, we showed that the expression pattern and regulation of TET1 isoforms are different and seems to be cell-of-origin and cell context dependent. This supports that further studies are needed to illuminate on the different functions of these isoforms in breast cancer initiation and progression and correlation with clinical outcomes.

## Data Availability Statement

The original contributions presented in the study are included in the article/**Supplementary Material**. Further inquiries can be directed to the corresponding author.

## Author Contributions

ZS generated the idea, designed research, data analysis, and wrote the paper. MA and AJ did the experimental work and analyzed the data. AK did the pathology work. All authors contributed to the article and approved the submitted version.

## Funding

Deutsche Forschungsgemeinschaft (DFG). Grant reference number BO 1743/7-1 to ZS.

## Conflict of Interest

The authors declare that the research was conducted in the absence of any commercial or financial relationships that could be construed as a potential conflict of interest.

## Publisher’s Note

All claims expressed in this article are solely those of the authors and do not necessarily represent those of their affiliated organizations, or those of the publisher, the editors and the reviewers. Any product that may be evaluated in this article, or claim that may be made by its manufacturer, is not guaranteed or endorsed by the publisher.

## Supplementary Material

Supplementary Figure 1 Validation of TET1 clones. **(A, B)** qRT-PCR on mRNA extracted from either MDA MB231 infected with *TET1* (TET1) or MDA MB231 infected with empty vector (EV) using either *TET1* specific primers **(A)** or primer pair where the forward primer targets HA-tag while the reverse primer targets *TET1* **(B)**. Results are shown after normalization to the level of the housekeeping gene *hUBC* and relative to mRNA levels in MDA MB231 control cells infected with empty vector. Bars represent SEM. *indicates that p-value is <0.05.
