# Supplementary figures and images for: TET1 Isoforms Have Distinct Expression Pattern, Localization and Regulation in Breast Cancer
Source: Front Oncol. 2022 May 12;12:848544. doi: 10.3389/fonc.2022.848544 (PMC9133332; doi:10.3389/fonc.2022.848544)

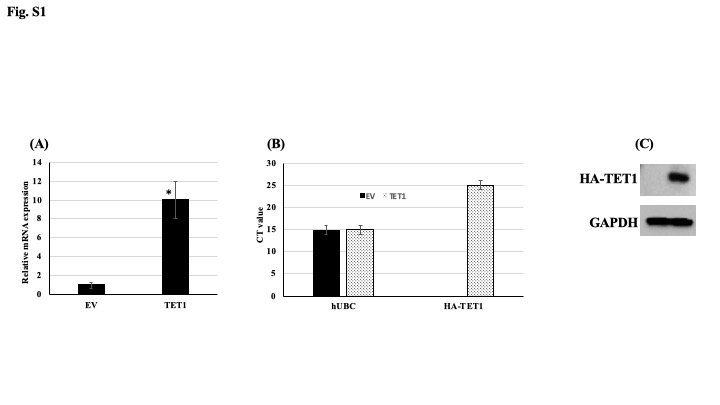

Supplement: Supplementary Figure 1 — Validation of TET1 clones. (A, B) qRT-PCR on mRNA extracted from either MDA MB231 infected with TET1 (TET1) or MDA MB231 infected with empty vector (EV) using either TET1 specific primers (A) or primer pair where the forward primer targets HA-tag while the reverse primer targets TET1 (B). Results are shown after normalization to the level of the housekeeping gene hUBC and relative to mRNA levels in MDA MB231 control cells infected with empty vector. Bars represent SEM. *indicates that p-value is <0.05. [file Image_1.jpeg]
