# Supplementary material for: TET1 Isoforms Have Distinct Expression Pattern, Localization and Regulation in Breast Cancer
Source: Front Oncol. 2022 May 12;12:848544. doi: 10.3389/fonc.2022.848544 (PMC9133332; doi:10.3389/fonc.2022.848544)
Supplement: Supplementary file 2 [file Table_1.docx]

**Table 1: List of primers used in real time PCR experiments for target genes**

| No | Gene | Primers | Reference |
| --- | --- | --- | --- |
| 1 | *AKT1* | F.P_5'- cacaaacgaggggagtacat -3' | NM_005163.2 |
|  |  | R.P-5'- tgcgccacagagaagttg-3' |  |
| 2 | *Cyclin B1* | F.P_5'- gtcaccaggaactcgaaaat -3' | NM_031966.3 |
|  |  | R.P-5'-ttaccaatgtccccaagagc-3' |  |
| 3 | *IDH 1* | F.P_5'- ctacatagctatgatttaggc -3' | NM_001282386.1 |
|  |  | R.P-5'- ctcaaccctcttctcatcagg-3' |  |
| 4 | *Nanog* | F.P_5'- acggagactgtctctcctct-3' | NM_024865.3 |
|  |  | R.P-5'- tttgcgacactcttctctgc-3' |  |
| 5 | *PCDH7* | F.P_5’-atggaaaatgattcaaggcctc-3’ | NM_001173523.1 |
|  |  | R.P_5’-aggctggctcttcttcctct-3’ |  |
| 6 | *SLIT2* | F.P_5’-gctatacaggcttgatctcagtg-3’ | NM_004787.4 |
|  |  | R.P_5’-ctgaatgccccatcttcaat-3’ |  |
| 7 | *Snail1* | F.P_5'- acactggcgagaagccctt -3' | NM_005985.3 |
|  |  | R.P-5'- gcctggcactggtacttctt -3' |  |
| 8 | *Wnt5A* | F.P_5'- atgaagaagtccattggaat -3' | NM_003392.4 |
|  |  | R.P-5'- ctgggcgaaggagaaaaata -3' |  |
| 9 | *hUBC* | F.P 5’- gtcgcagttcttgtttgtgg-3’ | NM_021009.6 |
|  |  | R.P 5’-gatggtgtcactgggctcaa-3’ |  |
| 10 | *TET1 exon10-11* | F.P_5’-ccacagggacattcacaaca-3’ | NM_030625.2 |
|  |  | R.P_5’-catggagctgctcatcttga-3’ |  |
| 11 | *HA-TET1* | F-HA_5’-gaggatacccctacgacgtg-3’ | NM_030625.2 |
|  |  | R.TET1_5’-tcccttggttgtctttcgtag-3’ |  |
